# Phylogenetic and whole genome analysis of first seven SARS-CoV-2 isolates in Bangladesh

**DOI:** 10.2217/fvl-2020-0201

**Published:** 2020-11-26

**Authors:** Nadim Sharif, Shuvra K Dey

**Affiliations:** ^1^Department of Microbiology, Jahangirnagar University, Savar, Dhaka 1342, Bangladesh

**Keywords:** Bangladesh, genome, mutation, SARS-CoV-2, spike protein, therapeutics, vaccine

## Abstract

**Aim:** Whole genome and peptide mutation analysis can specify effective vaccine and therapeutics against severe acute respiratory coronavirus-2 (SARS-CoV-2). **Materials & methods:** Whole genome similarity for Bangladeshi SARS-CoV-2 was determined using ClustalW and BLASTn. Phylogenetic analysis was conducted using neighbor-joining method. **Results:** 100% of isolates in Bangladesh were in the G clade. We found 99.98–100% sequence similarity among Bangladeshi isolates and isolates of England, Greece, USA, Saudi Arabia and India. Deletion of bases at 5′ untranslated region and 3′ untranslated region was detected. Substitution 261 (E→D) at NSP13 and 1109 (F→L) at spike (S) protein were detected. Substitution 377 (D→G) at nucleocapsid with common substitution 614 (D→G) at S were also detected. **Conclusion:** This study will provide baseline data for development of an effective vaccine or therapeutics against SARS-CoV-2.

## Graphical abstract

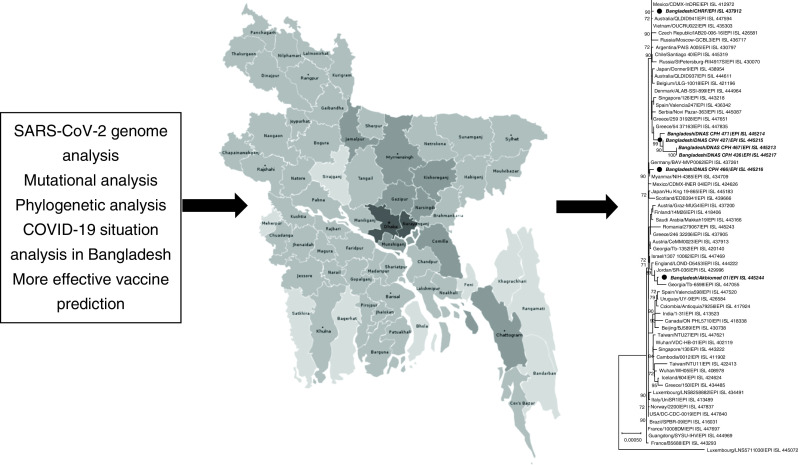

A novel species of coronavirus, severe acute respiratory coronavirus-2 (SARS-CoV-2) (family *Coronaviridae*), previously named as 2019-novel coronavirus (2019-nCoV), has emerged as a pandemic virus [1,2]. SARS-CoV-2 has caused the recent coronavirus disease-2019 (COVID-19) worldwide [3]. During the early 1960s, first human coronavirus 229E and human coronavirus OC43 were detected in patients having symptoms of pneumonia and common cold [4]. Other significant species from genera *Betacoronavirus* – SARS-CoV (2003), HCoV NL63 (2004), HKU1 (2005), MERS-CoV (2012) – have been reported to cause human respiratory system infection [1–3]. However, none of them transmitted globally as fast as SARS-CoV-2. About 7.3 million COVID-19 confirmed cases and 413,726 fatalities were reported from more than 210 countries within a short period of 6 months.

In December 2019, the first confirmed case of SARS-CoV-2 was reported from Wuhan, China. The case fatality rate increased from 2 to 7% globally [5]. Mode of transmission of SARS-CoV-2 includes contact (direct and indirect), droplets and fomites [6,7]. Primarily, SARS-CoV-2 infection causes respiratory tract illness with symptoms like SARS-CoV in human. COVID-19 patients develop significant clinical symptoms of the respiratory system [8]. Most common clinical features of COVID-19 are fever, cough, sore throat and shortness of breath [9,10]. Among newly evolving clinical features, chill, loss of taste or smell, feelings of shaking, headache, rash and muscle pain have appeared in a significant number of patients [9–11]. About 85% of the infected patients with mild symptoms have recovered. However, acute respiratory syndrome, acute pneumonia, difficulty in breathing, heart failure, kidney failure and failure of multiple organs have been detected in patients with severe COVID-19 [10–12].

SARS-CoV-2 is one of the largest enveloped RNA viruses. It is a nonsegmented, positive sense, ssRNA virus with a genome size of approximately 30,000 bases in length [2,12,13]. The genome also contains characteristics of a 5′ cap structure at the upstream region along with a 3′ poly (A) tail at the downstream region. Most frequently reported open-reading frames (ORFs) of SARS-CoV-2 genome are 1a, 1b, 3a, 3b, 6, 7a, 7b, 8a, 8b and 9b [14]. Of note, the first two ORFs of SARS-CoV-2 genome – 1a and 1b – occupy approximately 20,000 bases (two-third of genome) and encode for nonstructural (nsps) replicase proteins [12,15]. Among nsps, 16 nonstructural proteins – nsp1 to nsp16 – have been identified with defined functions. Four major structural proteins – spike (S), envelope (E), membrane (M) and nucleocapsid (N) are encoded by later ORFs (∼10,000 bases) [16]. The established genome order of coronavirus is 5′-leader-UTR-replicase-S-E-M-N-3′ UTR-poly (A) [12,15,17,18].

Variation in the whole genome of novel coronavirus is frequent [14]. The main aim of the study is to analyze the first seven whole genomes of SARS-CoV-2 in Bangladesh. This study aims to build baseline data on SARS-CoV-2 molecular epidemiology in Bangladesh. Another aim is to determine the evolutionary relationship of Bangladeshi SARS-CoV-2 by phylogenetic analysis. Mutational analysis will also be conducted to determine new or previous mutations that may affect the virus replication process, pathogenesis, proof reading mechanism, vaccine and therapeutic effectiveness.

## Materials & methods

### Data collection

Data were collected from different databases. Whole genomes of SARS-CoV-2 were collected from GISAID (www.gisaid.org) databases. COVID-19 cases and fatalities data were collected from Worldometers (www.worldometers.info/coronavirus), Johns Hopkins University COVID-19 database (https://coronavirus.jhu.edu/), Epidemiology, Disease Control and Research (www.iedcr.gov.bd/website/) website and Directorate General of Health Services (www.dghs.gov.bd/index.php/bd/) in Bangladesh website. Various environmental data were collected from Bangladesh Meteorological Department (http://live4.bmd.gov.bd/satelite/v/sat_infrared/) and AccuWeather (www.accuweather.com). Each month was divided into four equal weeks (W1–W4) except the last week (W5) contained 3 days in January, 1 day in February, 3 days in March, 2 days in April and 3 days in May, respectively. Appropriate institutional review board approval was taken from Biosafety, Biosecurity and Ethical Committee of Jahangirnagar University for this study. Approval number was BBEC, JU/M 2020/COVID-19/(10)1.

### Nucleotide sequence analysis

The nucleotide sequences of whole genome for SARS-CoV-2 were analyzed using Chromas 2.6.5 (Technelysium, Helensvale, Australia). Sequence homology was determined using the BLASTn program. Multiple sequence alignment was conducted in BioEdit 7.2.6 using the ClustalW Multiple Alignment algorithm [19]. Mutational analysis was performed for specific positions of SARS-CoV-2 whole genome and peptide chain.

### Phylogenetic & evolutionary relationship analysis

Phylogenetic and molecular evolutionary relationship analyses of Bangladeshi SARS-CoV-2 were conducted using the whole genome sequences of the references by the MEGA-X software [20]. Phylogenetic tree was generated with 1000 bootstrap replicates of the nucleotide datasets alignment. Neighbor-joining method was used for phylogenetic and molecular evolutionary analysis [21]. Kaimura-2 parameter was used for calculating the genetic distance. Sequence ID of Bangladeshi SARS-CoV-2 used in this study are provided in Table 1.

**Table 1.** Sequence ID of the first seven Bangladeshi SARS-CoV-2 genomes used in this study.

First seven Bangladeshi SARS-CoV-2 sequences ID
CHR|FEPI_ISL_437912
DNAS_CPH_467|EPI_ISL_445213
DNAS_CPH_471|EPI_ISL_445214
DNAS_CPH_427|EPI_ISL_445215
DNAS_CPH_466|EPI_ISL_445216
DNAS_CPH_436|EPI_ISL_445217
Akbiomed|EPI_ISL_445244

Reference sequences of SARS-CoV-2 from 58 countries were used for the phylogenetic analysis. GISAID references are: Mexico/CDMX-InDRE|EPI_ISL_412972, Luxembourg/LNS2299410|EPI_ISL_421745, United Arab Emirates/L0881|EPI_ISL_435125, Latvia|EPI_ISL_437090, Saudi Arabia/Jeddah66|EPI_ISL_437755, Saudi Arabia/Jeddah60|EPI_ISL_443182, Singapore/126|EPI_ISL_443218, USA/NY-NYUMC623|EPI_ISL_444740, Chile/Santiago_40|EPI_ISL_445319, Wales/PHWC-2B55B|EPI_ISL_445726, Germany/FFM3|EPI_ISL_447609, Greece/259_31928|EPI_ISL_447651, France/10008DM|EPI_ISL_447697, France/10003SN|EPI_ISL_447719, France/10006HC|EPI_ISL_447727, Greece/54_37163|EPI_ISL_447835, USA/DC-CDC-0019|EPI_ISL_447840, Denmark/ALAB-SSI-899|EPI_ISL_444964, Guangdong/SYSU-IHV|EPI_ISL_444969, Luxembourg/LNS5711030|EPI_ISL_445072, Serbia/Novi Pazar-363|EPI_ISL_445087, Japan/Hu_Kng_19-865|EPI_ISL_445183, Sweden/20-07741|EPI_ISL_445241, Romania/279067|EPI_ISL_445243, Chile/Santiago_80|EPI_ISL_445377, Wales/PHWC-2F065|EPI_ISL_446193, Thailand/Bangkok-0079|EPI_ISL_447020, Romania/279068|EPI_ISL_447054, Georgia/Tb-6598|EPI_ISL_447055, DRC/3653|EPI_ISL_447231, Israel/130710062|EPI_ISL_447469, Spain/Valencia597|EPI_ISL_447519, Spain/Valencia598|EPI_ISL_447520, India/CCMB_J278|EPI_ISL_447565, Australia/QLDID941|EPI_ISL_447594, Germany/FFM7|EPI_ISL_447613, Taiwan/NTU27|EPI_ISL_447621, Greece/238_31927|EPI_ISL_447645, France/10007LJ|EPI_ISL_447725, Greece/55_36015|EPI_ISL_447834, Greece/145_34726|EPI_ISL_447836, Norway/2200|EPI_ISL_447837, India/CCMB_K499|EPI_ISL_447865, Iceland/604|EPI_ISL_424624, Mexico/CDMX-INER_04|EPI_ISL_424626, Czech Republic/IAB20-006-16|EPI_ISL_426581, Uruguay/UY-9|EPI_ISL_426584, Spain/Madrid_H12_2902|EPI_ISL_428701, Russia/Moscow-77620|EPI_ISL_428889, Jordan/SR-036|EPI_ISL_429996, Russia/StPetersburg-RII4917S|EPI_ISL_430070, Beijing/BJ589|EPI_ISL_430738, Argentina/PAIS_A005|EPI_ISL_430797, Greece/150|EPI_ISL_434485, Luxembourg/LNS8258882|EPI_ISL_434491, Myanmar/NIH-4385|EPI_ISL_434709, Vietnam/OUCRU022|EPI_ISL_435303, Spain/Valencia247|EPI_ISL_436342, Russia/Moscow-GCBL3|EPI_ISL_436717, Italy/TE5543|EPI_ISL_436720, Latvia/017|EPI_ISL_437096, Indonesia/JKT-EIJK03|EPI_ISL_437191, Austria/Graz-MUG4|EPI_ISL_437200, Germany/BAV-MVP0062|EPI_ISL_437261, Turkey/HSGM-10232|EPI_ISL_437334, Poland/Wro-02|EPI_ISL_437625, Denmark/ALAB-SSI-137|EPI_ISL_437649, Saudi Arabia/Madinah258|EPI_ISL_437742, USA/WA-UW-6546|EPI_ISL_437860, Greece/246_32206|EPI_ISL_437905, Austria/CeMM0023|EPI_ISL_437913, Japan/Donner9|EPI_ISL_438954, Scotland/EDB3941|EPI_ISL_439666, Northern Ireland/NIRE-FAAED|EPI_ISL_441737, Saudi Arabia/Makkah19|EPI_ISL_443166, Singapore/130|EPI_ISL_443222, France/B5688|EPI_ISL_443293, England/LOND-D5453|EPI_ISL_444222, England/LOND-D606D|EPI_ISL_444272, Australia/QLDID937|EPI_ISL_444611, USA/NY-NYUMC610|EPI_ISL_444727, Wuhan/IVDC-HB-01|EPI_ISL_402119, Wuhan/WH05|EPI_ISL_408978, Cambodia/0012|EPI_ISL_411902, South Korea/SNU01|EPI_ISL_411929, Switzerland/1000477806|EPI_ISL_413024, Italy/UniSR1|EPI_ISL_413489, New Zealand/01|EPI_ISL_413490, South Korea/KUMC03|EPI_ISL_413513, India/1-31|EPI_ISL_413523, USA/CruiseA-24|EPI_ISL_414483, Hong Kong/VM20002582|EPI_ISL_414569, USA/WA-UW75|EPI_ISL_415603, Peru/010|EPI_ISL_415787, Brazil/SPBR-09|EPI_ISL_416031, Brazil/SPBR-10|EPI_ISL_416032, Iceland/242|EPI_ISL_417570, Colombia/Antioquia79256|EPI_ISL_417924, Portugal/PT0021|EPI_ISL_418006, Canada/ON_PHL3802|EPI_ISL_418337, Canada/ON_PHL5710|EPI_ISL_418338, Finland/14M26|EPI_ISL_418406, Germany/NRW-24|EPI_ISL_419541, Georgia/Tb-1352|EPI_ISL_420140, Belgium/ULG-10018|EPI_ISL_421196, Taiwan/NTU11|EPI_ISL_422413 and Netherlands/NA_163|EPI_ISL_422698.

## Results

### Demographic analysis

**Figure 1.**
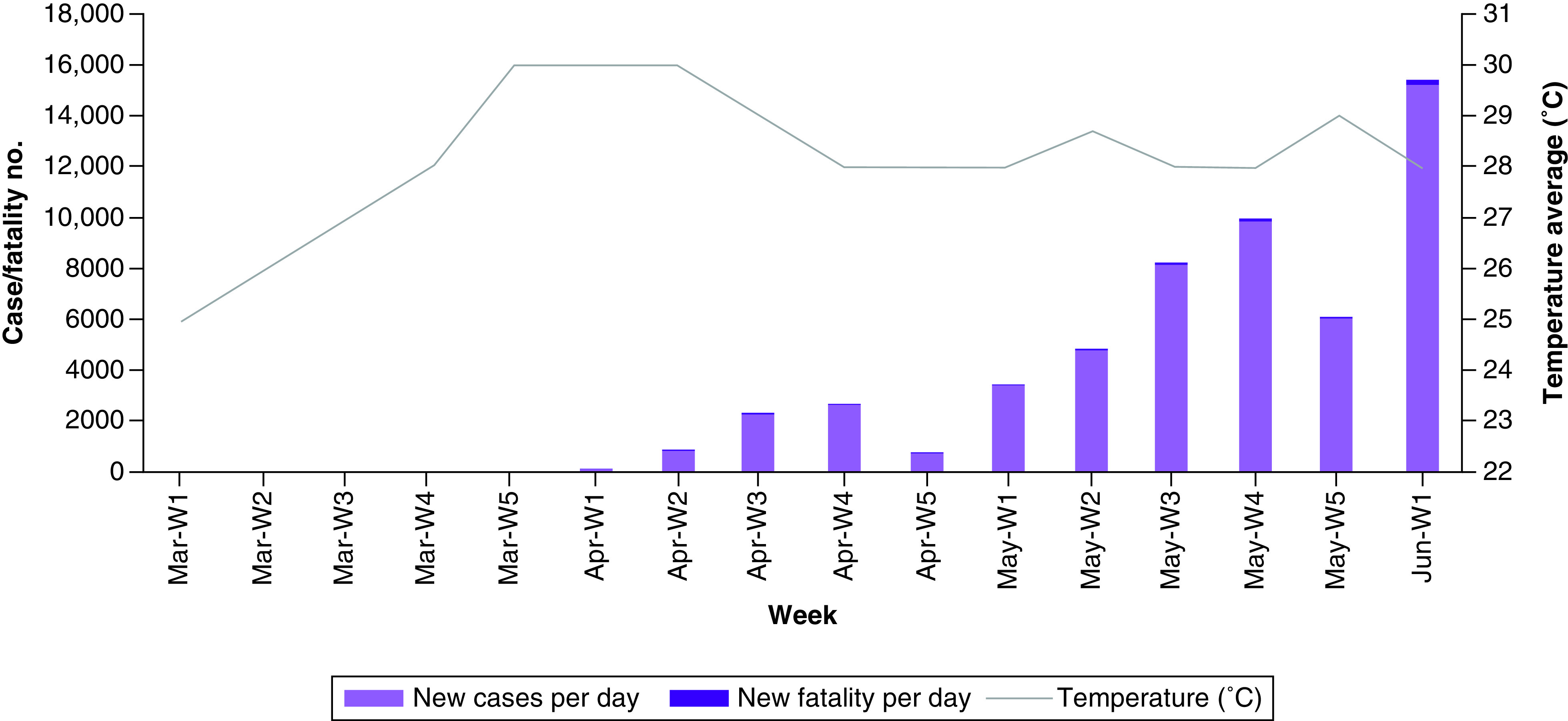
Weekly distribution of coronavirus disease-2019 cases and fatalities in relation with mean of average temperature per week in Dhaka, Bangladesh.

On 8 March 2020, the first COVID-19 case was detected in Bangladesh. Till 9 June, with 17% positive cases of total tests, about 71,675 confirmed cases and 975 fatalities were detected in Bangladesh. Most cases (∼35,000) had been reported from Dhaka, the capital of Bangladesh with an average increase rate of 5973 cases/week and 81 fatalities/week in the country. COVID-19 is increasing relatively slowly in Dhaka, which has a population density of 121,720/mi^2^. During 1 February 2020 to 9 June 2020, the minimum temperature average was 20°C, maximum temperature average was 32.5°C and mean temperature average was 26.5°C in Dhaka (Figure 1). Along with environmental factors, genotype variation is the main reason for a low number of COVID-19 cases compared with the expected number in Bangladesh. The first seven whole genomes of SARS-CoV-2 were sequenced from seven COVID-19 patients in Dhaka. Among these patients, 57% (four of seven) were male and 43% (three of seven) were female. Highest frequency (42.9%, three of seven) of SARS-CoV-2 was detected in age group 21–30 years, followed by 14.3% in 11–20 years, 28.6% in 31–40 years and 14.3% in 41–50 years, respectively.

### Phylogenetic & molecular evolutionary relationship analysis

**Figure 2.**
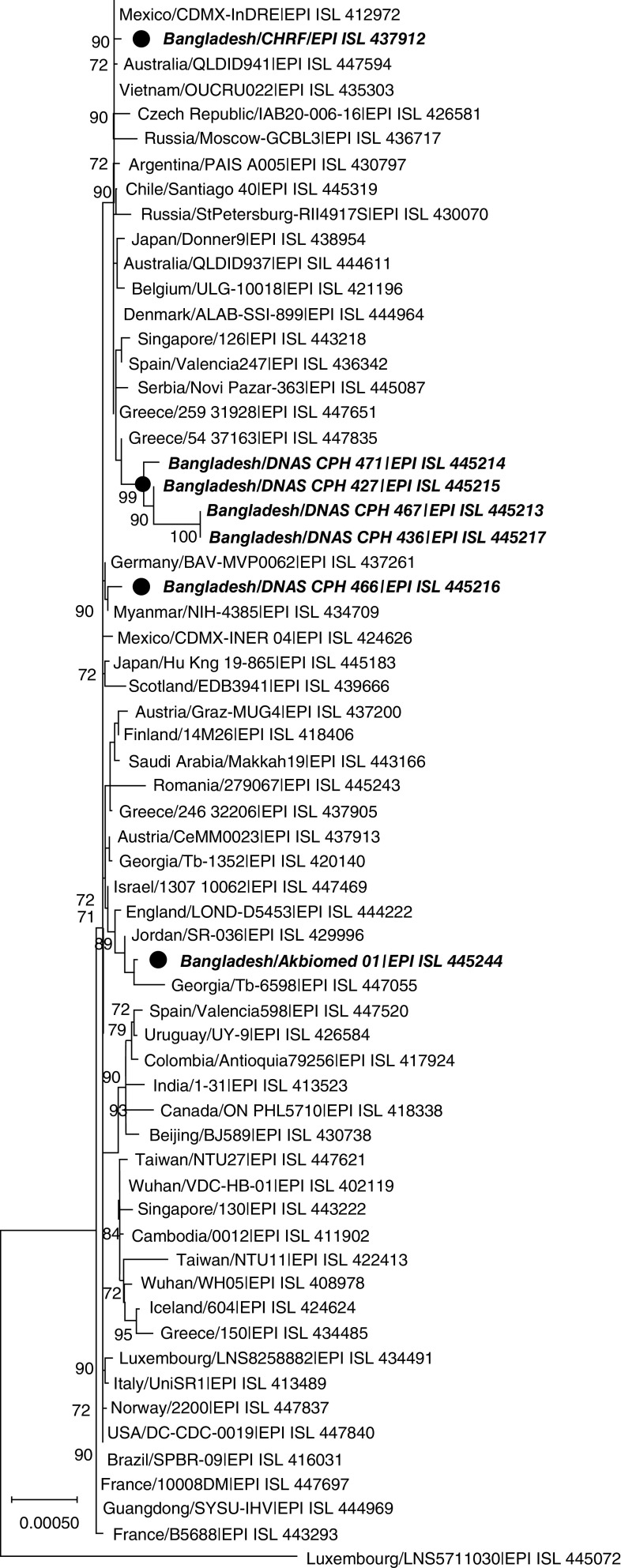
Phylogenetic tree of the first seven whole genome of severe acute respiratory coronavirus-2 in Bangladesh. The tree was constructed using whole genome of novel coronaviruses. Reference SARS-CoV-2 strains were selected from GISAID (accession numbers are indicated in the Materials & methods section). The scale indicates nucleotide substitutions per position. The numbers in the branches indicate the bootstrap values. Bangladeshi SARS-CoV-2 were highlighted in italic bold. SARS-CoV-2: Severe acute respiratory coronavirus-2.

The first seven sequenced SARS-CoV-2 in Bangladesh were from the G clade. Compared with 40,000 whole genomes, Bangladeshi SARS-CoV-2 were found to have 100–99.98% sequence similarity with reference sequences. The first sequenced whole genome in Bangladesh, Bangladesh/CHRF had 99.99% sequence similarity with whole genome of SARS-CoV-2 from Germany/FFM3, Sweden/20-07237, USA/NY-NYUMC623, Saudi Arabia/KAUST-Jeddah60, Latvia/011, United Arab Emirates/L0881 and Mexico/CDMX-InDRE_01 (Table 2). Another five whole genome sequences, namely, Bangladesh/DNAS_CPH_467, Bangladesh/DNAS_CPH_471, Bangladesh/DNAS_CPH_427, Bangladesh/DNAS_CPH_466 and Bangladesh/DNAS_CPH_436 had 99.98, 99.98, 99.98, 100 and 99.99% sequence similarity with USA/DC-CDC-0019, Wales/PHWC-2B55B, Germany/FFM3, Luxembourg/LNS2299410 and Greece/54_37163, respectively. Of note, these five sequences also shared about 99.99% sequence similarity with each other. Recently sequenced, Bangladesh/Akbiomed_01 had 99.98% sequence similarity with India/GBRC92 (Table 2). In phylogenetic analysis, CHRF|FEPI_ISL_437912 was closely related with SARS-CoV-2 from Latvia and UAE. Furthermore, DNAS_CPH_467|EPI_ISL_445213, DNAS_CPH_471|EPI_ISL_445214, DNAS_CPH_427|EPI_ISL_445215 and DNAS_CPH_436|EPI_ISL_445217 were closely related with each other and whole genome of Greece and Spain (Figure 2). Isolate DNAS_CPH_466|EPI_ISL_445216 was closely related with SARS-CoV-2 of Myanmar and England, while Akbiomed|EPI_ISL_445244 was related with isolates of Georgia and Jordan (Figure 2).

**Table 2.** Sequence similarity of Bangladeshi severe acute respiratory coronavirus-2 in relation with reference strains around the world.

Bangladeshi sequence	Reference sequence of SARS-CoV-2 genome	Similarity (%)
Bangladesh/CHRF|EPI_ISL_437912	Germany/FFM3/EPI_ISL_447609, Sweden/20-07237/EPI_ISL_445231, USA/NY-NYUMC623/EPI_ISL_444740, Saudi Arabia/KAUST-Jeddah60/EPI_ISL_443182, Saudi Arabia/KAUST-Jeddah66/EPI_ISL_437755, Latvia/011/EPI_ISL_437090, United Arab Emirates/L0881/EPI_ISL_435125, Mexico/CDMX-InDRE_01/EPI_ISL_412972	99.99
Bangladesh/DNAS_CPH_467|EPI_ISL_445213	USA/DC-CDC-0019/EPI_ISL_447840, Greece/54_37163/EPI_ISL_447835, Greece/263_32477/EPI_ISL_447653	99.98
Bangladesh/DNAS_CPH_471|EPI_ISL_445214	Wales/PHWC-2B55B/EPI_ISL_445726, Chile/Santiago_40/EPI_ISL_445319, Singapore/126/EPI_ISL_443218	99.98
Bangladesh/DNAS_CPH_427|EPI_ISL_445215	Germany/FFM3/EPI_ISL_447609, Chile/Santiago_40/EPI_ISL_445319	99.98
Bangladesh/DNAS_CPH_466|EPI_ISL_445216	Luxembourg/LNS2299410/EPI_ISL_421745, France/10006HC/EPI_ISL_447727, France/10002PM/EPI_ISL_447721, France/10003SN/EPI_ISL_447719, France/10008DM/EPI_ISL_447697	100
Bangladesh/DNAS_CPH_436|EPI_ISL_445217	USA/DC-CDC-0019/EPI_ISL_447840, Greece/54_37163/EPI_ISL_447835, Greece/259_31928/EPI_ISL_447651	99.99
Bangladesh/Akbiomed_01|EPI_ISL_445244	USA/DC-CDC-0019/EPI_ISL_447840, India/GBRC92/EPI_ISL_447554, India/GBRC70/EPI_ISL_447047, Wales/PHWC-2A835/EPI_ISL_445625, USA/NY-NYUMC539/EPI_ISL_444656	99.98

### Whole genome analysis of SARS-CoV-2

Whole genomes of the first seven SARS-CoV-2 in Bangladesh were analyzed. The first sequence, CHR|FEPI_ISL_437912, from Bangladesh was 29903 bases in length, sharing the highest similarity with the Wuhan reference sequences (NC_045512/Wuhan-Hu-1) by length. Another six whole genomes, DNAS_CPH_467|EPI_ISL_445213, DNAS_CPH_471|EPI_ISL_445214, DNAS_CPH_427|EPI_ISL_445215, DNAS_CPH_466|EPI_ISL_445216, DNAS_CPH_436|EPI_ISL_445217 and Akbiomed|EPI_ISL_445244, had sequence length of 29642, 29833, 29829, 29828, 29706 and 29823 bases, respectively. CHR|FEPI_ISL_437912 did not have any deletion mutation while the other six isolates had significant deletion at both ends. Furthermore, deletion of the first 25 bases at 5′ untranslated region (UTR) and 40–60 bases at 3′ UTR stem loop region were commonly detected in these six isolates. Besides, numbers of deletion mutations were detected in DNAS_CPH_467|EPI_ISL_445213 and DNAS_CPH_436|EPI_ISL_445217 after 27,000 bases. Insertion mutation was detected in only one whole genome, Akbiomed|EPI_ISL_445244, between position 202 and 203. Substitution point mutation was the most common in Bangladeshi SARS-CoV-2. At 5′ UTR, substitution 241 (C→T) was found in all the isolates from Bangladesh. Most frequently detected substitution point mutations at ORF1ab (266–21,555) region were 1163 (A→T), 3037 (C→T) and 14408 (C→T) in Bangladeshi SARS-CoV-2. In the spike protein-coding regions, substitution mutation 23403 (A→G) was frequent, while 24887 (T→C) was detected in only DNAS_CPH_427|EPI_ISL_445215. Large number of substitution and deletion were found at ORF7a, ORF7b and ORF8 (27394–28259) in DNAS_CPH_436|EPI_ISL_445217. Substitution point mutations 28881 (G→A), 28882 (G→A) and 28883 (G→C) in N protein region were common in most of the Bangladeshi isolates. Large number of substitutions and deletion mutations were also found at N protein (28274–29533), ORF10 (29558–29674) and 3′ UTR (29675–29903) regions in four genomes (Table 3).

**Table 3.** Mutational analysis of Bangladeshi severe acute respiratory coronavirus-2 whole genome with reference strain.

Isolates	5′ UTR (1–265)	ORF1ab (266–21555)	S protein (21563–25384)	ORF3a (25393–26220)	ORF7a (27394–27759), ORF7b (27756–27887)	N protein (28274–29533), ORF10 (29558–29674), 3′ UTR (29675–29903)
NC_045512/Wuhan-Hu-1 (Reference)	Reference	Reference	Reference	Reference	Reference	Reference
CHRF/EPI_ISL_437912	1 (A→N), **241 (C→T)**	**1163 (A→T), 3037 (C→T), 14408 (C→T)**, 17019 (G→T)	**23403 (A→G)**			**28881 (G→A), 28882 (G→A), 28883 (G→C)**
DNAS_CPH_467|EPI_ISL_445213	Deletion (1–25), **241 (C→T)**	**1163 (A→T), 3037 (C→T)**, 4444 (G→T), 8371 (G→T), 10979 (G→A), **14408 (C→T)**	**23403 (A→G)**	25609 (G→T)	Deletion (27435–27453), Deletion (27472–27672)	**28881 (G→A), 28882 (G→A), 28883 (G→C)**, 29403 (A→G), 29848 (T→A), 29849 (A→C), 29850 (A→T), 29852 (A→T), 29853 (G→A), 29855 (T→G), 29856 (T→C), 29857 (C→T), 29855 (T→C), Deletion (29859), 29880 (A→G), Deletion (29889–29903)
DNAS_CPH_471|EPI_ISL_445214	Deletion (1–25), **241 (C→T)**	**1163 (A→T)**, 2041 (T→C), **3037 (C→T)**, 4444 (G→T), 8371 (G→T), **14408 (C→T)**	**23403 (A→G)**			**28881 (G→A), 28882 (G→A), 28883 (G→C)**, 29403 (A→G), 29848 (T→A), 29850 (A→C), 29852 (A→T), 29853 (G→A), 29853 (C→A), 29855 (T→G), 29856 (T→C), 29857 (C→T), 29855 (T→C), Deletion (29859–29903)
DNAS_CPH_427/2020|EPI_ISL_445215	Deletion (1–25), **241 (C→T)**	**1163 (A→T), 3037 (C→T)**, 4444 (G→T), 8371 (G→T), **14408 (C→T)**	**23403 (A→G)**, 24887 (T→C)	25609 (G→T)		**28881 (G→A), 28882 (G→A), 28883 (G→C)**, 29403 (A→G) 29403 (A→G), 29848 (T→A), 29849 (A→C), 29850 (A→T), 29852 (A→T), 29853 (G→A), Deletion (29855–29903)
DNAS_CPH_466|EPI_ISL_445216	Deletion (1–25), **241 (C→T)**	**3037 (C→T)**, 3688 (C→T), **14408 (C→T)**, 15324 (C→T), 15895 (C→T)	**23403 (A→G)**			29848 (T→A), 29850 (A→C), 29852 (A→T), 29853 (G→A), Deletion (29854–29903)
DNAS_CPH_436|EPI_ISL_445217	Deletion (1–25), **241 (C→T)**	**1163 (A→T), 3037 (C→T)**, 4444 (G→T), 8371 (G→T), **14408 (C→T)**	**23403 (A→G)**	25609 (G→T)	27473 (C→A), 27475 (A→T), 27476 (C→T), 27478 (G→T), 27480 (A→C), 27481 (C→T), 27485 (T→C), Deletion (27482–27552), 27555 (T→G), Deletion (27563–27569), 27572 (G→A), 27578 (A→G), 27579 (A→C), 27581 (T→A), 27582 (T→C), 27583 (G→T), 27584 (C→T), 27585 (T→G), 27587 (T→G), 27588 (T→A), Deletion (27593–27609), 27610 (C→T), 27612 (C→T), 27613 (G→C), 27614 (T→A), 27618 (T→C), 27621 (G→A), 27622 (T→G), 27623 (T→A), 27624 (A→G), 27625 (C→T), 27629 (C→T), 27630 (C→T), Deletion (27634–27669), 27671 (T→G)	**28881 (G→A), 28882 (G→A), 28883 (G→C)**, 29403 (A→G), 29848 (T→A), 29849 (A→C), 29850 (A→T), 29852 (A→T), 29853 (G→A), 29855 (T→G), 29856 (T→C), 29857 (C→T), 29855 (T→C), Deletion (29859–29903)
Akbiomed_01/2020|EPI_ISL_445244	Deletion (1–25), 26 (A→T), 27 (A→T), 28 (C→A), 29 (A→G), 30 (A→G), 31 (A→C), 198 (G→A), 199 (G→A), G (202–203), **241 (C→T)**	**3037 (C→T), 14408 (C→T)**	**23403 (A→G)**			29794 (G→C), Deletion (29848–29903)

Bold letter indicates most common among Bangladeshi isolates.

ORF: Open-reading frame; UTR: Untranslated region.

In amino acid peptide sequence, significant and rare point mutations were found in Bangladeshi novel coronavirus genomes. At NSP2 protein, 120 (I→F) was detected for the first time in SARS-CoV-2 genome. While at NSP3, 1184 (Q→H) was very rare and detected in Bangladeshi isolates. At NSP6, 3 (V→M) was detected for the first time in DNAS_CPH_467/2020|EPI_ISL_445213 globally. Furthermore, at NSP12, 323 (P→L) was found in all of the Bangladeshi isolates. However, at NSP13, 261 (E→D) was detected in CHRF/EPI_ISL_437912. Spike proteins common mutation 614 (D→G) was present in every isolate from Bangladesh. Of note, 1109 (F→L) at S protein was detected for the first time in DNAS_CPH_471/2020|EPI_ISL_445214 worldwide. At NS3, 172 (G→C) was detected in some of the Bangladeshi isolates. Furthermore, at N protein, 203 (R→K), 204 (G→R) and rare 377 (D→G) were detected in some of isolates (Table 4).

**Table 4.** Analysis of new/rare substitution point mutations in the peptide chain of Bangladeshi novel coronavirus isolates with reference strains.

Sequence ID	Protein Amino acid position										
	NSP2	NSP3	NSP6	NSP12	NSP13	S		NS3	N		
120	1884	3	323	261	614	1109	172	203	204	377	
Wuhan/WIV04/2019 (GISAID reference)	I	Q	V	P	E	D	F	G	R	G	D
NC_045512/Wuhan-Hu-1 (NCBI reference)	I	Q	V	P	E	D	F	G	R	G	D
CHRF/EPI_ISL_437912	**F**	Q	V	L	**D**	G	F	G	K	R	D
DNAS_CPH_467|EPI_ISL_445213	**F**	**H**	**M**	L	E	G	F	**C**	K	R	**G**
DNAS_CPH_471|EPI_ISL_445214	**F**	**H**	V	L	E	G	**L**	G	K	R	**G**
DNAS_CPH_427|EPI_ISL_445215	**F**	**H**	V	L	E	G	F	**C**	R	G	D
DNAS_CPH_466|EPI_ISL_445216	I	Q	V	L	E	G	F	G	R	G	D
DNAS_CPH_436|EPI_ISL_445217	**F**	**H**	V	L	E	G	F	**C**	K	R	**G**
Akbiomed_01|EPI_ISL_445244	I	Q	V	L	E	G	F	G	R	G	D

Bold letter indicates first time/rare.

## Discussion

The novel coronavirus has triggered the ongoing COVID-19 pandemic by infecting over 7.2 million people worldwide [1]. Like other beta coronaviruses, SARS-CoV-2 is also acquiring mutations in its genome and evolving rapidly [14,22]. Whole genome analysis of SARS-CoV-2 in any region will be essential to understand the infection dynamics of COVID-19. The whole genome of Bangladeshi SARS-CoV-2 has been sequenced recently. In comparison to regions with lower temperature, both case numbers and fatalities are less in Bangladesh. The circulating SARS-CoV-2 isolates in Bangladesh are less deadly than those of the USA and Europe [22].

The first seven isolates in Bangladesh were in the G clade. Among the first seven isolates, 57% (four of seven) were detected in male and 43% (three of seven) female. Furthermore, the highest percentage (42.9%, three of seven) of SARS-CoV-2 in Bangladesh was detected in patients of 21–30 years, followed by 28.6% in 31–40 years, 14.3% in 11–20 years and 14.3% in 41–50 years, respectively. The distribution of gender was similar with previous studies in Europe, China and Asia, but age distribution of COVID-19 patients in Bangladesh was unique [1,23,24].

The phylogenetic analysis revealed that the first sequenced SARS-CoV-2 in Bangladesh CHRF|EPI_ISL_437912 was closely related with beta coronavirus from the UAE, Latvia, Saudi Arabia, Mexico and the USA and clustered with them. In the BLAST analysis of CHRF|EPI_ISL_437912, this study detected 99.99% sequence similarity with Germany/EPI_ISL_447609, Sweden/EPI_ISL_445231, USA/NY/EPI_ISL_444740, Saudi Arabia/Jeddah60/EPI_ISL_443182, Latvia/EPI_ISL_437090, United Arab Emirates/EPI_ISL_435125 and Mexico/EPI_ISL_412972. This indicates the probable evolutionary linkage of this isolate with European, Middle East and American beta coronaviruses. Another four of these isolates, DNAS_CPH_467|EPI_ISL_445213, DNAS_CPH_471|EPI_ISL_445214, DNAS_CPH_427|EPI_ISL_445215 and DNAS_CPH_436|EPI_ISL_445217 clustered with each other and were closely related with coronavirus of Greece and Spain in the phylogenetic tree. Of note, DNAS_CPH_466|EPI_ISL_445216 clustered with coronavirus of England and Myanmar, and this cluster was closely related with isolates of France and Germany as well. Bangladeshi isolates DNAS_CPH_467|EPI_ISL_445213, DNAS_CPH_471|EPI_ISL_445214 and DNAS_CPH_427|EPI_ISL_445215 shared 99.98% sequence identity with USA/EPI_ISL_447840, Greece/EPI_ISL_447835, Wales/EPI_ISL_445726, Chile/EPI_ISL_445319 and Germany/EPI_ISL_447609 in BLAST analysis. Furthermore, isolate DNAS_CPH_466|EPI_ISL_445216 was found to have 100% sequence similarity with Luxembourg/EPI_ISL_421745 and France/EPI_ISL_447727, while isolate DNAS_CPH_436|EPI_ISL_445217 had 99.99% similarity with USA/EPI_ISL_447840 and Greece/EPI_ISL_447835. Isolate Akbiomed|EPI_ISL_445244 clustered with beta coronavirus of Georgia, Jordan and England in the phylogenetic tree, while shared 99.99% sequence similarity with USA/EPI_ISL_447840, India/EPI_ISL_447554 and India/EPI_ISL_447047 in BLAST. This study detected that Bangladeshi SARS-CoV-2 had significant evolutionary relationships with European, American and Asian SARS-CoV-2.

Whole genome analysis of novel coronavirus is necessary to understand its infectivity, fatality associated with specific variants and to predict any alteration of efficacy of possible drug or vaccine due to target proteins modification of the virus [25,26]. In whole genome analysis, we detected unique and new point mutations in Bangladeshi novel coronavirus isolates. Numbers of sequences from 5′ UTR (1–265) and 3′ UTR (29675–29903) regions were missing for six isolates in Bangladesh. Both 5′ and 3′ ends of the coronavirus are important for regulatory functions of the genome [27]. Deletion at 5′ UTR (1–25) of Bangladeshi isolates confirmed the deletion of stem loop-1 (SL1) (7–31) regions that might cause defective *cis*-acting elements interaction during the virus replication and RNA synthesis. Besides 5′ UTR (1–25) deletion, Akbiomed|EPI_ISL_445244 isolate had a number of point synonymous mutations at SL1, SL5A and SL5B regions with one insertion of G between 202 and 203 base in the SL5A region. Furthermore, substitution point mutation 241 (C→T) was common in all Bangladeshi isolates. Deletion and substitution mutations at SL5A and SL5B of 5′ UTR are involved in altered efficiency of coronavirus replication and infection pattern by changing interaction of the genome with viral nucleocapsid protein (N) and nsp1 protein [27]. Of note, at 3′ UTR (29675–29903) regions, substitution point mutation and deletion were common in SL regions in six Bangladeshi isolates. Along with 5′ UTR stem loop structure, 3′ UTR stem loop structure regulates viral replication as a *cis*-acting elements [27]. Alteration or deletion of sequences at 3′ UTR (29675–29903) in Bangladeshi isolates indicates changes of coronavirus replication strategy in these regions.

In the protein-coding regions, significant substitution point mutation was detected in Bangladeshi isolates. At ORF1ab (266–21555) regions, 1163 (A→T), 3037 (C→T) and 14408 (C→T) were frequent in Bangladeshi isolates. Substitution of isoleucine with phenylalanine at NSP2 120 (I→F) was detected in this study [14,22]. Another substitution mutation at papain like protease, NSP3 1884 (Q→H), glutamine to histidine, in Bangladeshi isolates was the first to be detected worldwide [14,22]. Substitution of valine by methionine at replicase nonstructural protein (NSP6), 3 (V→M) was reported from DNAS_CPH_467/2020|EPI_ISL_445213 in Bangladesh [14,22]. Furthermore, substitution at NSP12, 323 (P→L), one of the common mutations at RNA dependent RNA polymerase region (RdRp), was also detected in seven isolates. Of note, substitution of glutamate by aspartate at helicase peptide region NSP13 261 (E→D) was detected in CHRF/EPI_ISL_437912 in Bangladesh [14,22]. Substitution at ORF1ab has been reported from coronavirus worldwide [14,22]. However, different new substitutions at NSP2, NSP3 (papain-like protease), NSP6 (replicase nonstructural protein), NSP12 (RdRp) and NSP13 (helicase) in Bangladeshi isolates reported in this study are involved in altered replication efficiency, peptide processing capability, autophagy strategy and proof reading mechanism during genome duplication [14,22,28,29]. Importantly, we also detected substitution mutations at spike protein regions in Bangladeshi coronavirus isolates. At spike protein, 614 (D→G) substitution of aspartate with glycine was detected in all Bangladeshi isolates that had been previously reported to be associated with high case fatality in Europe [30]. In DNAS_CPH_471|EPI_ISL_445214, substitution of phenylalanine with leucine at 1109 (F→L) of spike protein was detected in this study. This unique mutation at spike protein may affect the receptor binding and also neutralizing antibody binding with virus particles.

Substitution mutation at 25609 (G→T) of ORF3a was detected in three of the seven isolates. Mutation at ORF3a is most frequent in Europe followed by Asia, Oceania and North America, respectively [22,24]. Alteration of bases at ORF3a regions is also crucial as peptide of this portion is involved with T-cell-mediated immunity [26]. In isolate DNAS_CPH_467|EPI_ISL_445213, deletion at ORF7a region was detected while numerous point mutations and deletion were detected at ORF7a and ORF7b in isolate DNAS_CPH_436|EPI_ISL_445217. Mutation in accessory protein ORF7a can lead to several altered pathogenic processes including apoptosis of host cell and inhibition of cellular protein synthesis [30–32]. In three of seven Bangladeshi isolates, substitution of glycine with cysteine was detected at 172 (G→C) in accessory protein NS3 that had been unique and reported for the first time in England. Furthermore, two frequent point mutation at 203 (R→K) and 204 (G→R) and one rare point mutation at 377 (D→G) in the nucleocapsid (N) region were detected in Bangladeshi isolates. First two mutations of N protein have been reported mostly from England and countries of Europe and the third one has been reported for only ten-times including Bangladeshi isolates [14,22].

Specific mutations, both deletion and substitution, in Bangladeshi coronavirus isolates were detected at various multiple sites in the genome and in the peptide chain. Of note, several new mutations at ORF1ab regions, specifically in the RdRp and its accessory proteins, will allow the virus to multiply without proof reading that will increase the possibility of accumulating more new mutations in the genome. Furthermore, along with a previous mutation associated with high case fatality, a new mutation at spike protein was also detected that increases the virus’ chance of escaping antibodies or drugs targeting the spike protein. To the best of our knowledge, this is one of the first studies to report phylogenetic and genomic analysis of the first seven sequenced novel coronavirus in Bangladesh. This study reported significant new mutations at important sites in the novel coronavirus genome and antigenic peptide regions. These mutations will affect virus replication strategy and antigenic properties that will ultimately change the virus capability to infect and help the virus to escape from antibodies and drugs. This study will be the baseline database of coronavirus genome analysis that will help to predict effective vaccine and drug targets of coronavirus.

## Conclusion

In a pandemic like COVID-19, whole genome analysis of the pathogen is important in order to understand the transmission and severity of the disease accurately. With limited resources, the number of whole genome analysis in Bangladesh is lower than in other developing countries. This study investigated the total mutation, phylogeny and evolution of the first seven whole genomes of SARS-CoV-2 in Bangladesh. They were closely related with each other and isolates from Germany, the USA, Saudi Arabia, France, Greece and India. Acquisition of unique mutations along with common mutations throughout the genome suggested rapid change of the circulating strains in Bangladesh. This study will provide a baseline to whole genome research of novel coronaviruses in Bangladesh.

**Summary points**

- Approximately 57% (four of seven) severe acute respiratory coronavirus-2 (SARS-CoV-2) isolates were detected in male and 43% (three of seven) in female patients.
- Approximately 100% Bangladeshi SARS-CoV-2 shared 99.98–100% sequence similarity with isolates of Germany, Sweden, the USA, Saudi Arabia, England, Myanmar and India.
- Deletion of bases at 5′ untranslated region and 3′ untranslated region in Bangladeshi isolates was specified.
- New substitution 1109 (F→L) with deadly substitution 614 (D→G) at spike protein was detected.
- Mutations at RNA-dependent RNA polymerase regions may lead to accumulation of random mutations in the novel coronavirus genome in future.
